# Early Detection of Food Safety and Spoilage Incidents Based on Live Microbiome Profiling and PMA-qPCR Monitoring of Indicators

**DOI:** 10.3390/foods13152459

**Published:** 2024-08-03

**Authors:** May Cohen Hakmon, Keren Buhnik-Rosenblau, Hila Hanani, Hila Korach-Rechtman, Dagan Mor, Erez Etkin, Yechezkel Kashi

**Affiliations:** 1 Department of Biotechnology and Food Engineering, Technion—Israel Institute of Technology, Haifa 3200003, Israel; mayc@campus.technion.ac.il (M.C.H.); buhnik@bfe.technion.ac.il (K.B.-R.); hilah@bfe.technion.ac.il (H.H.); rechtman.hila@gmail.com (H.K.-R.); 2 Gene-G Ltd., Kfar Tavor 1524100, Israel; daganmor14@gmail.com; 3 Maadaney Yehiam (1993) Ltd., Kibbutz Yehiam 2512500, Israel; erez@yehiam.com

**Keywords:** microbiome profile signature, rapid pathogen detection, defective food production, pastrami, lactate deficiency, genus-specific qPCR, PMA-qPCR

## Abstract

The early detection of spoilage microorganisms and food pathogens is of paramount importance in food production systems. We propose a novel strategy for the early detection of food production defects, harnessing the product microbiome. We hypothesize that by establishing microbiome datasets of proper and defective batches, indicator bacteria signaling production errors can be identified and targeted for rapid quantification as part of routine practice. Using the production process of pastrami as a model, we characterized its live microbiome profiles throughout the production stages and in the final product, using propidium monoazide treatment followed by 16S rDNA sequencing. Pastrami demonstrated product-specific and consistent microbiome profiles predominated by *Serratia* and *Vibrionimonas*, with distinct microbial signatures across the production stages. Based on the established microbiome dataset, we were able to detect shifts in the microbiome profile of a defective batch produced under lactate deficiency. The most substantial changes were observed as increased relative abundances of *Vibrio* and *Lactobacillus*, which were subsequently defined as potential lactate-deficiency indicators. PMA-qPCR efficiently detected increased levels of these species, thus proving useful in rapidly pinpointing the production defect. This approach offers the possibility of the in-house detection of defective production events with same-day results, promoting safer food production systems.

## 1. Introduction

Food safety is a significant concern in the modern world. Millions of people annually suffer from foodborne illnesses due to the consumption of unsafe food [1,2,3]. The increased shelf life of products, consumption of ready-to-eat foods, and a growing number of high-risk consumers further aggravate the abundance of foodborne illness outbreaks [1,2,3]. Along with food pathogens, spoilage microorganisms have garnered further interest for their damage to food quality. Even though food spoilage may not directly contribute to food outbreaks, some spoilage microorganisms produce toxins and are also considered opportunistic human pathogens [4]. Foodborne illness outbreaks and their related recalls have significant consequences, not only for the consumers, but also for the manufacturers. Recalls drive massive amounts of food loss [5], decreased purchase intentions, as well as legal and reputational damage [6,7]. These negative impacts are further exacerbated by heightened public attention due to the availability of information through the media. Taken together, these consequences translate to enormous economic losses for the food industry. To reduce or avoid food-related outbreaks and recalls, rapid routine testing for pathogenic and spoilage microorganisms is of high importance [5]. However, the stringent requirement to identify as little as one colony-forming unit (CFU) in a food sample due to low infective doses of some of the pathogens poses a significant challenge for food safety and regulatory bodies.

To meet the sensitivity requirements, the majority of microbial food-safety tests are based on culture-based techniques, which are time-consuming, taking at least 24 h and sometimes more than a week to complete [8]. Numerous new analytical methods are being introduced to the food testing market, including biochemical, spectroscopic, immunological, protein-based, and DNA-based technologies [9]. However, in order to enable the required sensitivity, these methods require pre-enrichment steps and therefore are not fast enough to allow the same-day detection of microbiological hazards [10,11]. This calls for the development of novel strategies enabling the sensitive and rapid detection of pathogens and food-spoilage microorganisms during the early stages of food production and before marketing. 

Here, we assessed the use of a novel strategy harnessing the product’s microbiome profile to rapidly detect defective production events. Microbiome profiling is a common method for characterizing complex microbial communities inhabiting a specific environment. Recently, it has been increasingly applied in the food-production niche [12,13,14,15]. Given the dynamic nature of bacterial communities and their rapid adaptation to environmental changes [16], we hypothesized that the microbiome profile of a food product should deviate from its typical signature upon encountering defective production events. Furthermore, we postulated that the deviating taxa could serve as early-warning molecular indicators that may be quantified using rapid assays as part of a routine practice, signaling potential disruptions or anomalies in the production process. We therefore evaluated whether such an approach could be employed to detect irregular events in the production line of barbecued (BarBQ) pastrami. BarBQ pastrami was chosen as the model product due to its multi-stage process, which begins with a high microbial load feedstock, which ultimately has to meet high safety and quality standards at the final product level and throughout its shelf life. A protocol for differentiating live and dead bacteria in the pastrami product enabled characterizing the live microbiome profiles of BarBQ pastrami along the production chain. We demonstrated the ability to differ between microbiome profiles of a normal and invalidated product batch and to identify specific bacterial indicators for a defective production event. These indicators then served as targets for specific PMA-qPCR tests and were demonstrated as effective in detecting the shifts in bacterial indicators quantities, enabling rapid distinguishing between the proper and non-valid batches. The use of microbiome profiling to construct a dataset and identify bacterial indicators, followed by the routine assessment of indicators carried out on site, enables the obtainment of testing results and the identification of improper batches within a few hours. 

## 2. Materials and Methods

### 2.1. Pastrami Sampling and DNA Extraction 

All sampling procedures were conducted at the Yehiam Delicatessen meat plant between 2019 and 2022. The sampling of production-line surfaces (cutting knives, conveyor belt, conveyor belt surroundings, and workers’ gloves), two to three samples of each, was carried out by swabbing 100-cm^2^ surface areas with 3M™ Sponge-Sticks and 10 mL neutralizing buffer (3M™, Saint Paul, MN, USA), which were compatible with the cleaning agents used on the production line. The swabs were suspended in 10 mL neutralizing buffer which was supplied with the Sponge-Sticks; these were cooled immediately after sampling and processed within 4 h. Samples of 5 mL were subjected to DNA extraction using QIAamp BiOstic Bacteremia DNA Kit (12240-50; Qiagen, Hilden, Germany) [17], according to the manufacturer’s instructions. The microbiome characterization of the meat products (Table S2) was targeted to 4–5 samples taken from different product regions and collected from independent batches. Analysis was carried out on four batches of BarBQ pastrami (composed of chicken and turkey), one batch of smoked pastrami (composed of chicken and turkey; containing different spices than the BarBQ pastrami), three batches of Kabanos (thin, dry sausage composed of chicken, turkey, and beef) and three batches of chicken sausage. All batches of each product were produced within a single month, while the first BarBQ pastrami batch was produced 8 months earlier than the other three. Smoked pastrami has the same meat content as BarBQ pastrami but is produced through a different production process. 

The microbiome characterization of the BarBQ pastrami along the production line was carried out by analyzing samples collected from the different production stages of a single batch (5 samples for each step; raw material: uncooked minced chicken and turkey meat; brine: water, maltodextrin, potato starch, 2% salt, soy protein, dextrose, potassium lactate, sodium diacetate, carrageenan, E451 phosphate, cellulose fibers, nutritional fibers, sodium erythorbate, sodium nitrate, flavorings and aromas; mixture: raw material + brine; product after cooking: pastrami product after the cooking step; product after slicing: the final cooked product, thinly sliced). The microbiome of potassium lactate-deficient BarBQ pastrami was characterized by analyzing samples collected along the different production stages of two parallel batches produced at a large scale with and without potassium lactate. All of the collected products were immediately cooled and processed within 20 h. For the lactate-deficient experiment, products with and without potassium lactate were further kept at 4 °C and DNA was extracted at 1, 2, 6, and 13 weeks. Sample processing included the slicing of 4–5 samples from the product taken from different sites. Samples were then subjected to DNA extraction. 

A preliminary assessment of different methods for microbial meat sampling (stomaching, rinsing, surface swabbing or surface scraping [18] showed that meat rinsing was the best in terms of the absence of PCR inhibition. Based on that, the working protocol included the rinsing of 4 to 8 g of sliced meat product or a 6 mL brine sample in 20 mL Ringer’s solution (BR0052G; Thermo Fisher Scientific, Waltham, MA, USA) in a 50 mL Falcon tube for 20 min at room temperature, rotated at a speed of 100 RPM. This was followed by centrifugation (200× *g*, 1 min) to remove crude material. The supernatant (10 mL) was filtered through a 40 µm nylon cell strainer (Falcon, Cary, NC, USA) and centrifuged (1000× *g*, 10 min). A 1 mL aliquot of the supernatant (or 10^6^ CFU of live or dead *Escherichia coli* suspended in lysogeny broth [LB], or 250 ng pure DNA, as tested in the framework of the propidium monoazide [PMA] setup; see Supplementary Material Section) was subjected to PMA treatment to facilitate the PCR amplification of live cells as described in [19] with minor modifications. Briefly, 2.5 µL of 20 µM PMA (Biotium, Fremont, CA, USA) solution was added to the bacterial suspensions, which were incubated for 10 min in the dark, followed by 20 min incubation under 5 W royal-blue LEDs (LEDsupply, Randolph, VT, USA). DNA extraction was further carried out using the MagMAX^TM^ CORE Nucleic Acid Purification Kit (A32702; Thermo Fisher Scientific) [20] according to the manufacturer’s instructions with slight modifications. Briefly, samples were centrifuged (8000× *g*, 5 min) and 700 µL of the upper supernatant was discarded. The remaining 300 µL was mixed with 100 µL PK solution (90 µL PK buffer for MagMax, 10 µL MagMax CORE proteinase K), vortexed, and incubated (55 °C, 30 min). This was followed by an additional centrifugation step (15,000× *g*, 2 min) and the mixing of 250 µL of the supernatant with 20 µL MagMax CORE magnetic beads. Lysis/binding solution (350 µL MagMax CORE lysis solution + 350 µL MagMax CORE binding solution for a total 700 µL) was then added, and the samples were vortexed for 10 min, followed by centrifugation at a slightly higher speed (17,000× *g*, 2 min; the increase in centrifugation speed facilitates bead separation in all types of samples, including the oily solutions mainly observed for raw material or meat–brine mixtures). The supernatant was then decanted, and the beads were washed consecutively with 500 µL each of MagMax CORE wash solutions 1 and 2, and dried. The DNA was eluted with 50 µL of MagMax CORE elution buffer after a 10 min vortexing step. DNA was stored at −20 °C pending analysis.

### 2.2. Microbiome Profiling 

DNA samples were sequenced at the Technion Genomics Center (TGC), Haifa, Israel, using Illumina MiSeq. Briefly, the DNA concentration was measured using Qubit Flex (Invitrogen, Waltham, MA, USA) with the Qubit dsDNA HS Assay Kit (Q32854; Invitrogen). The 16S libraries were constructed simultaneously according to Illumina 16S Metagenomic Sequencing Library Preparation using primers, amplifying the 16S V3 and V4 region located at positions 341 bp and 805 bp (F341: 5′-TCGTCGGCAGCGTCAGATGTGTATAAGAGACAGCCTACGGGNGGCWGCAG-3′; R805:5′-GTCTCGTGGGCTCGGAGATGTGTATAAGAGACAGGACTACHVGGGTATCTAATCC-3′; each containing the appropriate overhang adapter sequence: forward overhang—5′-TCGTCGGCAGCGTCAGATGTGTATAAGAGACAG-3′ [locus-specific sequence]; reverse overhang—5′-GTCTCGTGGGCTCGGAGATGTGTATAAGAGACAG-3′ [locus-specific sequence]). The original Illumina protocol was adjusted to begin with a 1.25 ng DNA input, allowing the analysis of all samples, including those with low DNA concentrations. The number of PCR cycles at the first PCR stage was therefore increased, with the PCR conditions as follows: 1 cycle at 95 °C for 3 min; 30 cycles at 95 °C for 30 s, 55 °C for 30 s, 72 °C for 30 s; and a final cycle of 72 °C for 5 min. Library quality control was performed by measuring the library concentration using Qubit dsDNA HS Assay Kit (Q32854; Invitrogen) and size determination was performed using the TapeStation 4200 with the D1000 Kit (5067-5582; Agilent Technologies, Santa Clara, CA, USA). All libraries were mixed in a single tube at equal molarity. The sequencing data were generated on the Illumina Miseq with 250 paired-end reads.

Quantitative Insights Into Microbial Ecology (QIIME2) [21] version 2-2023.5.1 was used for demultiplexing based on sample-specific barcode sequences, allowing the assignment of reads to their respective samples. Quality filtering was further performed to remove low-quality reads (with scores below Q25) and to improve the overall reliability of the dataset. Primer sequence removal, length trimming, and chimera removal were carried out using the DADA2 plugin (version 2023.5.0). Taxonomy was assigned against the Silva database (v138) as the reference [22] and further used to infer taxa bar plots.

### 2.3. PMA-qPCR 

*E. coli* primers targeted to the *uidA* gene (F: 5′-GCAGTTTCATCAATCACCAC-3′ R: 5′-CTCCTACCGTACCTCGCATTAC-3′), as well as *Vibrio-* and *Lactobacillus*-specific primers (*Vibrio*_F: 5′-GGCGTAAAGCGCATGCAGGT-3′, *Vibrio*_R: 5′-GAAATTCTACCCCCCTCTACAG-3′ [23]; *Lactobacillus*_F: 5′-TGGAAACAGRTGCTAATACCG-3′, *Lactobacillus*_R: 5′-GTCCATTGTGGAAGATTCCC-3′ [24]) or universal primers (F: 5′-GCAGGCCTAACACATGCAAGTC-3′ R: 5′-CTGCTGCCTCCCGTAGGAGT-3′) targeted to the 16S rRNA gene were used to quantify bacterial abundance in the DNA samples following PMA treatment. *Vibrio* and *Lactobacillus* have been chosen as indicators based on microbiome profiling data demonstrating deviations in their relative abundances between proper and defective batches. Each reaction mixture was composed of 5 µL of Fast SYBR Green Master Mix (Applied Biosystems, Thermo Fisher Scientific, Waltham, MA, USA), 0.3 µL of each primer, 2.4 µL DDW, and 2 µL DNA sample at either 1:1 or 1:10 dilution to overcome possible PCR inhibitors in the samples. The qPCRs were conducted in the Step One Plus Real-Time PCR System (Applied Biosystems) as follows: 1 cycle at 95 °C for 10 min, followed by 40 cycles at 95 °C for 30 s and 60 °C for 1 min. A post-amplification melting curve was further generated, and the results were analyzed using StepOne Software v2.3 (Applied Biosystems). Calibration curves were generated using DNA extracted (MagMAX CORE Nucleic Acid Purification Kit; A32702; Thermo Fisher Scientific) from pure *E. coli* ATCC 8739, *Vibrio cholera* O9, and *Lactobacillus plantarum* DSM 20174 cultures grown in LB or De Man–Rogosa–Sharpe (MRS) medium. Curves were generated in 10^2^ to 10^8^ CFU/mL ranges to assess the cell concentration range for which efficient amplification is demonstrated. For *Vibrio-* and *Lactobacillus*-specific qPCRs, primer specificity was confirmed by a lack of amplification in the qPCR that was targeting DNA extracted from representatives of the different taxa detected as part of the pastrami bacterial population (*Serratia marcescens* ATCC14756, *Pseudomonas fluorescens* ATCC13525, and *Listeria innocua* ATCC33090); additionally, as negative controls, *Vibrio*-specific qPCRs were confirmed by a lack of amplification in the qPCR targeted to the DNA of *Lactobacillus plantarum* DSM 20174, while *Lactobacillus*-specific qPCRs were confirmed by a lack of product in the qPCR targeted to the DNA of *Vibrio cholera* O9. Amplification reactions were followed by melting-curve analysis. Artificial bacterial communities composed of these different taxa in known quantities were subjected to genus-specific PMA-qPCR to verify specific and correct quantifications. 

### 2.4. Statistical Analysis

Alpha diversities were calculated using Shannon’s entropy. Beta diversities were determined by computing weighted UniFrac distance and were subjected to principal coordinate analysis (PCoA). 

Significant differences in community composition among different samples were determined by a permutational multivariate analysis of variance (PERMANOVA) test or by the analysis of similarities (ANOSIM) using QIIME2. 

Differences in the levels of total bacteria, *Lactobacillus*, *Vibrio*, or *E. coli*, as well as in Shannon indices, were determined using one-way analysis of variance (ANOVA), followed by means of separation through Tukey’s honestly significant difference (HSD) test or via *t*-test (two-tailed) using the statistical software JMP 16 (2021 version, SAS Institute Inc., Cary, NC, USA). Significantly different groups (α = 0.05), as determined by the Tukey test, are indicated.

## 3. Results

### 3.1. Characterization of the Pastrami Microbiome

This study aimed to assess the use of the microbial signature of a food product as an early warning measure for food quality and safety, using BarBQ pastrami as the model product. The microbiome analysis of BarBQ pastrami must consider that a substantial portion of the microbiome consists of dead bacterial cells as a consequence of the thermal treatment stage, which is integral to the production process. Therefore, we implemented a protocol to distinguish between live and dead bacteria in the microbiome-profiling process. The protocol takes advantage of PMA’s ability to penetrate and bind to the DNA of dead cells selectively, thereby preventing its amplification (see description in Supplementary Section; Table S1A and Table S1B). 

**Figure 1:**
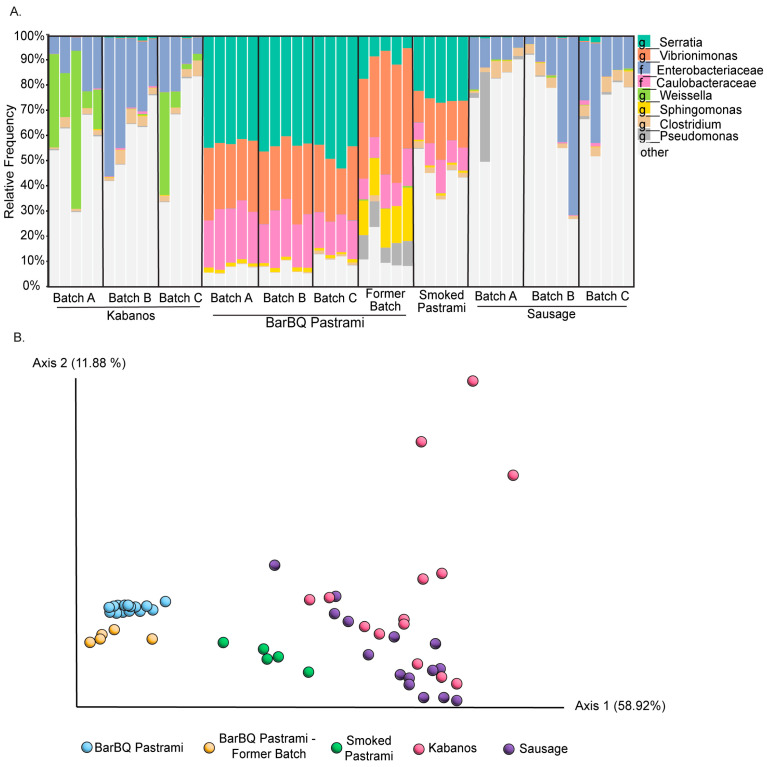
Live microbiome profiling of different meat products and batches. Microbiome composition in the different meat products was assessed using PMA treatment followed by 16S rRNA gene sequencing. (**A**) Relative abundances displayed by taxa bar plots. Each column represents the live microbiome of a single sample with the eight most-abundant genera listed on the right side of the diagram. (**B**) Beta diversities of bacterial communities clustered using PCoA, based on weighted UniFrac measure; significant differences were observed among the microbiota profiles of the different products (ANOSIM R = 0.7).

In the next stage, we defined the types of samples for analysis in the project’s scope, as the pastrami-related microbiome can be analyzed directly in the product or the meat production surfaces. Our preliminary data indicated relatively wide variation and low uniformity for the bacterial profiles obtained from the tested production surfaces, as sample replicate from the same surface demonstrated different profiles. This was in contrast to the pastrami meat products, which tended to present much more consistent and reproducible bacterial profiles (Figure 1A,B); these were, therefore, chosen as the sampling target for the study. Beyond microbiome uniformity among samples of the same batch, we further assessed whether microbiome uniformity is kept amongst different batches and whether the microbiome signature specifically characterizes the product. The products’ microbiome profile assessment was based on a survey that included three Kabanos (semi-dried sausage) batches, three chicken sausage batches, four BarBQ pastrami batches, and another batch of a different pastrami product (smoked pastrami), with four to five samples processed and analyzed for each batch. Significant differences were observed among the microbiota profiles of the different products (ANOSIM, R = 0.7), with the BarBQ pastrami batches presenting a distinct and highly consistent microbiome profile compared to those of the other products (Figure 1A,B). The BarBQ pastrami microbiome further demonstrated significantly lower bacterial richness compared to the other tested products (Table 1), with *Serratia* and *Vibrionimonas* predominating (Figure 1A). 

A general stability in the microbiome profiles of the BarBQ pastrami batches was observed, with Beta diversities of bacterial communities clustering together (Figure 1B). The microbiome profiles of the three batches sampled within a single month (batches A–C) clustered tightly compared to that of the former batch produced 8 months earlier, which was slightly shifted along the PC2 axis (Figure 1B). This variation might have been due to time, material, or sampling issues, and dataset adjustments should be included in the routine practice. Despite the general stability among batches and their tight clustering, differences among them were still observed, even amongst the adjacent batches (*q* = 0.016 between batches A and C or between the former batch and batch A/B; *q* = 0.038 between batches B and C; *q* = 0.014 between the former batch and batch C). This encourages the construction of a microbiome dataset of increased sizes to overcome batch-to-batch variation. A comparison of all BarBQ pastrami batches to batches of the other products revealed much wider differences among products (Figure 1A,B). The microbiome profiles of the BarBQ pastrami batches were distinct, not only from those of Kabanos composed of a different raw material (chicken, turkey and beef mixture; *q* < 0.001), but also from that of chicken sausages (*q* < 0.001) and the smoked pastrami product (*q* < 0.001). The latter is produced from the same feedstock as the BarBQ pastrami but is subjected to different treatments and contains different spices. This suggests that not only the feedstock type but also the production procedure and added spices are reflected in the microbiome profile of the product.

**Figure 2:**
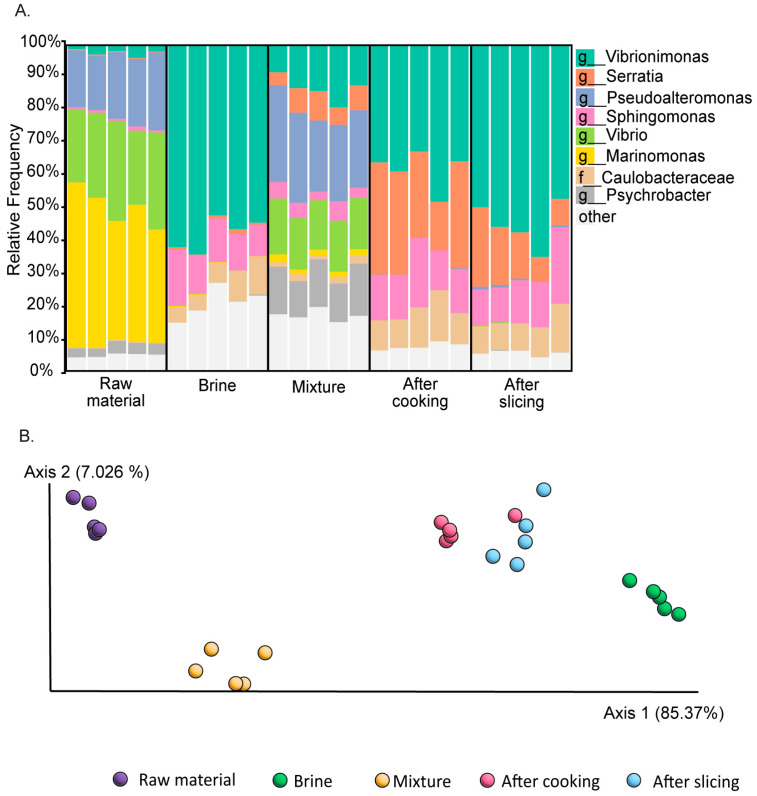
The live microbiome profiling of main stages along the BarBQ pastrami production chain. The microbiome composition of the live bacteria in the different production stages was assessed using 16S rRNA gene sequencing. (**A**) Relative abundances displayed by taxa bar plots. Each column represents the live microbiome of a single sample with the eight most-abundant genera listed on the right side of the diagram. (**B**) Beta diversities of bacterial communities clustered using PCoA, based on weighted UniFrac measure; ANOSIM R = 0.96; *p* < 0.05 for comparison of every two stages. Raw material: raw chicken and turkey meat after defrosting and grinding. Brine: a mixture of water, spices and preservatives. Mixture: raw material mixed with the brine, incubated overnight at 4 °C. After cooking: mixture cooked for 3 h to an internal Tmax of 72 °C. After slicing: the final product sliced into thin slices and packed.

Following the demonstration of stable and product-specific bacterial signatures, and the creation of a dataset which enables monitoring deviations from the normal profile at the final product level, we prepared the groundwork for recognizing microbiome profile deviations during the production process as well. The BarBQ pastrami-production process is initiated by mixing raw ground chicken and turkey meats with brine containing water, spices, and preservatives. The mixture is stored at 4 °C overnight, cooked for 3 h to an internal Tmax of 72 °C, cooled to 4 °C, and sliced and packaged. As each stage may be a source for imperfect production events, we characterized the microbiome signatures along the production stages of a properly produced product. The analysis revealed a typical bacterial signature for each production stage, with high variation in the microbiome profiles across the different production stages (ANOSIM, R = 0.96; *p* < 0.05 for comparison of every two stages; Figure 2A,B), underscoring the unique microbial composition associated with each phase of production.

The raw meat and brine samples exhibited distinct profiles, reflected in a merged microbiome profile of the raw meat–brine mixture samples. Notably, the mixture samples contained a higher relative abundance of the *Psychrobacter* genus compared to the raw meat and brine samples. This observation suggests that the overnight incubation at 4 °C selectively favored the growth of psychrophilic bacteria, consequently altering the microbiome profile. The cooking process, which has a major effect on the live bacterial content in the product, was associated with a remarkable change in the microbiome profile. The bacterial richness of post-cooking samples decreased markedly compared to pre-cooking samples (Table 2). Furthermore, the relative abundance of several taxa, including *Psychrobacter* [25] and *Vibrio* [26], decreased after the cooking stage, while the abundance of several other genera increased, with *Vibrionimonas* [27] being the most prevalent. The relative abundances of the predominant taxa remained relatively stable after the cooking stage and throughout the subsequent slicing process, despite the latter being prone to contamination. Overall, the analysis revealed a typical bacterial signature for each production stage.

**Figure 3:**
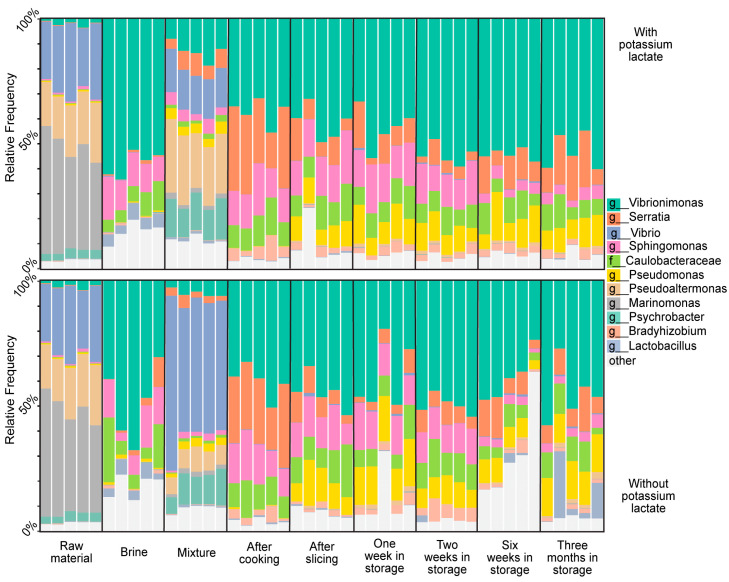
The live microbiome profiling of main stages along the pastrami production chain and a long-term storage period, with and without potassium lactate. The microbiome composition in the different production stages of the properly produced batch (upper plot) and the lactate-deficient batch (lower plot) was assessed using 16S rRNA gene sequencing. Each column represents the live microbiome of a single sample with the 11 most-abundant genera listed on the right side of the diagram. Raw material: raw chicken and turkey meat after defrosting and grinding. Brine: a mixture of water, spices and preservatives. Mixture: raw material mixed with the brine, incubated overnight at 4 °C. After cooking: mixture cooked for 3 h to an internal Tmax of 72 °C. After slicing: the final product sliced into thin slices and packed. One/two/six weeks/three months in storage represent the long-term storage periods of the products at 4 °C.

Based on this gathered knowledge and the bacterial signature dataset for a properly produced BarBQ pastrami batch, we conducted microbiome profiling under a defective production run. Our hypothesis was that we would observe substantial shifts in the microbiome profiles of a defective batch when compared to the proper batch, either during the production process or in the final product. Most parameters along the pastrami-production process, such as product temperature during cooking or cooling, are closely monitored to minimize defective production events. One of the parameters that is difficult to automatically monitor and therefore prone to human error is the addition of preservatives such as potassium lactate to the brine. We therefore challenged the pastrami microbiome profile in a defective batch deliberately produced without potassium lactate and compared it to that of a properly manufactured batch containing ~1% potassium lactate (both batches also contained sodium diacetate and sodium nitrite as additional preservatives). The live microbiome profiles of the two batches were assessed during the production process and for a further 3 months of shelf life at 4 °C. Five samples were processed and analyzed for each of the production stages. Overall reproducible microbiome patterns were obtained among replicates, representing the different production stages of the two batches (Figure 3). However, sample reproducibility during the shelf-life period was substantially poorer for the defective batch, indicating less control of the lactate-free product’s microbial population over time. The absence of potassium lactate was further linked to a notable shift in the relative abundances of specific taxa. Most prominently, there was an increased proportion of live *Vibrio* in the meat–brine mixture stage (Figure 3) [26]. Alongside the increase in the proportion of *Vibrio*, we identified an increase in the relative abundance of lactobacilli during the long-term storage period, as compared to the properly manufactured batch (Figure 3). These shifts in bacterial composition were accompanied by parallel shifts in bacterial richness. Lactate deficiency was correlated with decreased richness at the meat–brine mixture stage, which may indicate the niche occupation of specific taxa (such as *Vibrio*) under these conditions. During the long-term storage, however, lactate deficiency was correlated with increased bacterial richness, indicating different population dynamics along the shelf life of lactate-deficient samples (Table 3).

With the *Vibrio* and *Lactobacillus* genera in hand as candidates for early-warning indicators for out-of-standard production, we turned to testing the use of a rapid quantification assay specifically targeting each of these indicator taxa [26,28] with the aim of detecting changes in their quantities in a rapid manner. 

### 3.2. Rapid Detection of Vibrio and Lactobacillus as Potential Bacterial Indicators Based on PMA-qPCR

**Figure 4:**
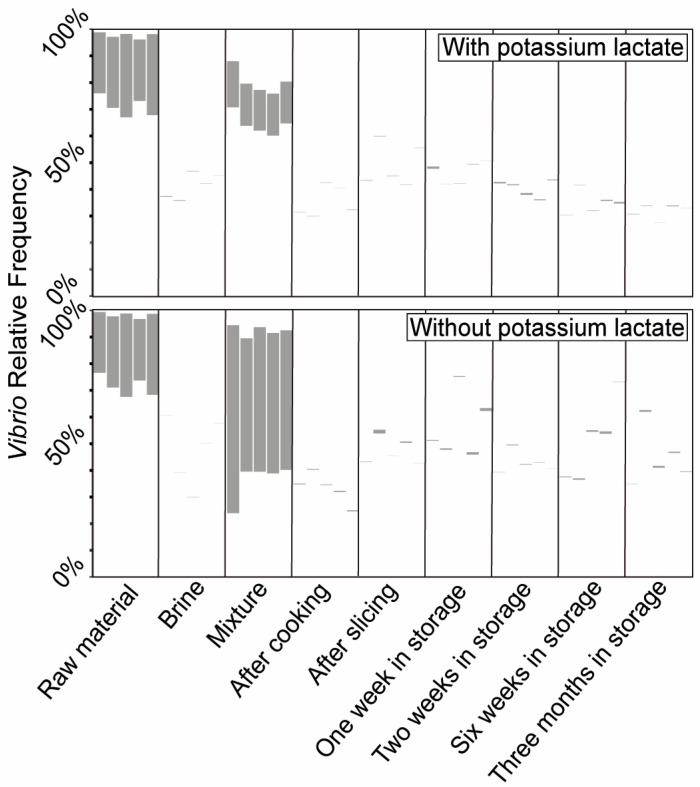
Highlighted *Vibrio* prevalence along different stages of the pastrami production process and a following long-term storage period, with and without potassium lactate. The relative abundance of *Vibrio* out of the entire microbiome composition, obtained for the different production stages of the properly produced batch (**upper plot**) and the lactate-deficient batch (**lower plot**), corresponds to the one presented in Figure 3.

**Figure 5:**
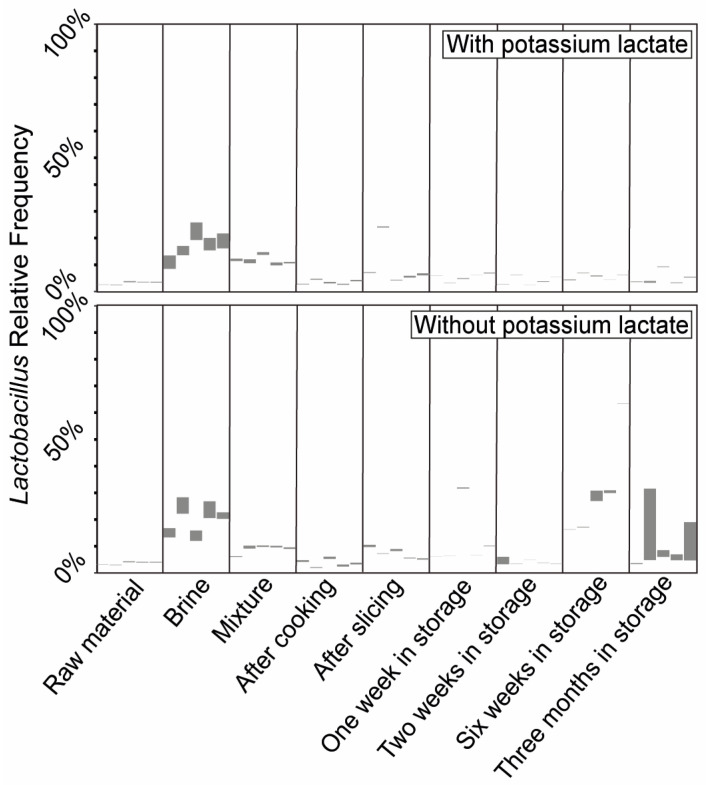
Highlighted *Lactobacillus* prevalence along different stages of the pastrami production process and a following long-term storage period, with and without potassium lactate. The relative abundance of *Lactobacillus* out of the entire microbiome composition, obtained for the different production stages of the properly produced batch (**upper plot**) and the lactate-deficient batch (**lower plot**), corresponds to the one presented in Figure 3.

Based on our thorough pastrami microbiome characterization, we designed rapid identification assays targeting the candidate indicator taxa, which may be used for the routine assessment and early detection of defective production events. PMA-qPCR assays were applied to quantify the levels of the exemplary potential indicators, namely *Vibrio* spp. in the meat–brine mixture and lactobacilli after packaging. Each indicator was quantified on the same set of DNA samples that had been used for the live microbiome analysis (Figure 3). *Vibrio*-specific PMA-qPCR demonstrated significantly lower *Vibrio* levels in the meat–brine mixture that contained lactate, as compared to the mixture without lactate, or to the raw material (Figure 4 and Table 4). This indicates that *Vibrio* indeed proliferated in the meat–brine mixture without lactate. The post-cooking stages of both proper and lactate-deficient batches showed reduced *Vibrio* levels compared to the pre-cooking stages by at least one order of magnitude, indicating that the cooking stage efficiently decreased *Vibrio* levels in both batches. This also points to the potential of applying *Vibrio* PMA-qPCR tests to indicate inadequate cooking and for the cross-contamination of the products after the cooking stage. *Lactobacillus*-specific PMA-qPCR (Figure 5 and Table 5) demonstrated increased levels of the genus during the long-term storage of the lactate-deficient batch at 4 °C, as opposed to the proper batch with *Lactobacillus* levels below the detection limit. *Lactobacillus* was detected in the raw material, decreased below the detection limit following the production process (tested after slicing), and rose again during storage under lactate deficiency. The PMA-qPCR results further clarified that the increase in the relative abundances of *Vibrio* or lactobacilli under lactate deficiency, as observed using the microbiome analysis, results from the actual growth of these bacteria. 

Overall, the calibrated PMA-qPCR assays were useful to promptly identify microbial fluctuations linked with a defective or out-of-standard production process.

The gathered results, therefore, imply the feasibility of rapid quantification assays targeted to specific indicators that are chosen based on a microbiome dataset for the sensitive early-warning detection of safety and quality-related events.

## 4. Discussion

Food manufacturers are constantly facing significant food safety and quality challenges [29]. A failure to meet current stringent food testing standards may lead to foodborne crises and product recalls, resulting in severe health-related consequences and substantial financial losses [5,7]. Therefore, early-warning platforms detecting deviations from quality and safety standards during production are invaluable.

In this study, the microbiomes of food products were analyzed to gain a deeper understanding of microbial dynamics during the food-production process and to assess the potential of food microbiomes to correlate with deviations from the normal production process and indicate defective and out-of-standard production events. Focusing on processed meat as a model, we analyzed its live microbiome by selectively preventing the DNA amplification of dead bacterial cells using PMA treatment (Ref. [19] and Supplementary Material Section). This is crucial when studying heat-treated food products as they contain a substantial portion of dead bacterial cells [30]. While the entire microbiome comprising both live and dead bacteria is more reflective of the historical record of the product’s microbial composition, the live microbiome is dynamic and rapidly adapting, containing bacterial taxa that may serve as indicators for out-of-standard batches.

With the tool of live microbiome analysis in hand, we first defined the target samples to be analyzed in the scope of the study, as the product-related microbiome may be reflected by the product itself and by the plant’s processing surfaces. While accumulating studies are expressing interest in the latter [13,15,31], our preliminary data indicated low uniformity in the bacterial profiles obtained for the tested production surfaces. This calls for deepening the characterization and constructing larger datasets as a base for the better mapping of the production-line environment in a follow-up study. On the other hand, microbiome analysis targeted at the meat products revealed product-specific signatures, fulfilling an essential requirement for using the microbiome as a potentially reliable indicator set. 

Further focus was put on the production of BarBQ pastrami, our model product, which presented unique and reproducible bacterial profiles. Alongside the product-specific bacterial signatures and the high consistency observed among BarBQ pastrami batches, batch-to-batch variations still appeared. This encourages the construction of a dataset of increased size, with routine periodical sampling to fine-tune the data. The predominant live bacteria in BarBQ pastrami appeared to be *Vibrionimonas* and *Serratia*; the latter has indeed been found in various natural environments, including food products of plant and animal origin [32]. Other known genera linked with pastrami spoilage (*Lactobacillus* and sulfate-reducing bacteria such as *Desulfovibrio*) [33] have been further identified at substantially lower levels. 

Beyond establishing the bacterial signatures of the final product as stable and product-specific, we further demonstrated the production of stage-specific bacterial signatures along the BarBQ pastrami production process. Raw meat–brine mixture samples presented a relatively high abundance of the *Psychrobacter* genus, possibly reflecting the overnight incubation of the mixture under psychrophilic (4 °C) conditions. Moreover, as expected, a substantial shift in the microbiome profile was demonstrated following the cooking step, reflecting a variation in the heat resistance of the different bacterial taxa. The shift may also have been somewhat affected by different technical aspects, such as a variation in the accessibility of the PMA reagent to different cell types following heat treatment. Either way, applying the same microbiome profiling protocol on samples from different stages along the production chain resulted in a typical bacterial signature for each stage. Among the taxa that deviated most prominently following the heat treatment were *Psychrobacter* [25] and *Vibrio* [26], both presenting decreased abundances, reflecting their heat sensitivity. The two are linked with the pastrami-production environment in negative contexts: *Psychrobacter* is well-known as a part of the spoilage microorganisms of chilled proteinaceous foods [25], and *Vibrio* includes potentially pathogenic species such as *V. cholerea*, *V. vulnificus,* and *V. parahaemolyticus* which can cause foodborne illness [26]. Their prevalence following heat treatment may indicate inadequate heat treatment or a cross-contamination of the pastrami among the production stages. Both scenarios can introduce harmful bacteria into the final product, which is a routine concern to food producers, highlighting the importance of early warning food safety detection. 

With a full microbiome database in hand, reflecting a properly produced BarBQ pastrami product along the production process and at the final product level, we tested whether defective production events correlate with deviations in the relative abundances of specific bacterial taxa. We analyzed the bacterial signatures of the product under the deficiency of potassium lactate, a commonly used preservative in meat products. Lactate salts prevent the growth of pathogenic bacteria and spoilage microorganisms and are further used to enhance and maintain the appearance, texture, color, and tenderness of the meat during storage [34]. Lactate addition is difficult to monitor automatically, and this step is therefore prone to human error. Indeed, a lack of potassium lactate was correlated with weakened control over the microbial population, as the bacterial signature’s reproducibility was poorer during the product’s shelf life for the defective batch compared to the normal batch. Furthermore, lactate deficiency was associated with shifts in the relative abundances of specific taxa. The proportion of live *Vibrio* in the meat–brine mixture increased, as was further detected using PMA-qPCR. This might be a result of the higher viability of some of the *Vibrio* members under cold temperatures, allowing them to grow rapidly in the cold, protein-rich substrate [26] during the overnight incubation step at 4 °C and under lactate deficiency. The increase in *Vibrio* abundance in the meat–brine mixture allowed us to mark this step as an optional hazard analysis and critical control point (HACCP) and encouraged us to apply *Vibrio* quantification at the meat–brine mixture stage as an early-warning tool for lactate deficiency.

An additional shift associated with lactate depletion was an increase in the relative abundance of lactobacilli during the long-term storage of the product, which was further detected using PMA-qPCR. Indeed, once preservative levels decrease, the highly prevalent lactobacilli are expected to be among the first to spoil the pastrami product [35,36]. The high diversity of the genus *Lactobacillus*, with its wide range of metabolic properties, enables its persistence in a variety of food products [28]. Moreover, lactobacilli are known to tolerate the added nitrite preservative [37], proliferate in anaerobic environments, and spoil the product by causing defects such as sour off-flavors, discoloration, gas production, slime production, and decreased pH [38]. Therefore, *Lactobacillus* quantification aids in assessing product quality during storage and was further demonstrated here to signal improper production. 

The comprehensive microbiome analysis carried out here provided a deep understanding of the expected taxa distribution in the product, their dynamics, and the factors that shape and control them along the production process. This promotes the identification of HACCPs and sources of optional contamination along the production process. Importantly, the microbiome signatures of similar products may vary when produced in different regions and under varied production processes. Therefore, it is crucial to construct a microbiome database specific to each product and production facility. This ensures that the database accurately reflects the unique microbial profiles associated with particular products and their specific manufacturing environments. Based on the microbiome datasets, which should be updated and adjusted by periodical microbiome analyses, indicator taxa can be identified based on a comparison of the microbiome profiles developed in a proper production process to those developed in defective batches. Once indicators have been identified, routine assays should be designed for their rapid quantification to identify out-of-standard batches. In our study, we employed PMA-qPCR assays to specifically target and quantify *Vibrio* and *Lactobacillus* genera that were identified as potential indicators for lactate deficiency. Unlike microbiome analysis, which infers the relative abundance of taxa within a sample, PMA-qPCR provides the precise quantification of each target bacterial species. Consequently, the results from these two analytical approaches may not fully align. Despite their inherent differences as well as the variation in technical aspects, such as PCR conditions and primer sequences, the observations made with the PMA-qPCR assays closely aligned with those of the microbiome analysis. The increased levels of the two indicators demonstrated using PMA-qPCR indicate an excessive growth of *Vibrio* spp. in the meat–brine mixture and lactobacilli after packaging in the lactate-deficient batch. Together with previous work suggesting the use of PMA treatment and downstream qPCR analysis for accurate bacterial quantification in similar foods [39], the use of PMA-qPCR is suggested as a reliable-indicator quantification assay during the production process. 

In general, such proposed DNA-based assays for bacterial indicators offer significant advantages over current methods for detecting safety and quality issues in food products. These methods could theoretically be utilized to identify specific bacterial deviations associated with any production process, stage, or type of defect. However, the entire methodology will need to be considered specifically for each plant and product, as several technical factors may impede their dissemination. In cases where there is high variability in raw materials or in the production process itself, inconsistent microbial signatures may arise during routine analysis. This inconsistency can hinder the creation of a reproducible microbiome database that accurately reflects a properly produced product. Overall, the reproducibility and replicability of the approach must be assessed for specific products, manufacturing, and industrial scales. 

Once the methodology has been successfully validated for a specific product, it should provide rapid results within hours and eliminate the need for enrichment steps required as part of existing methods, which support pathogen growth and are typically prohibited in food plants. Therefore, the suggested assays can be conducted in-house, avoiding the need to send samples to external laboratories. The streamlined approach enables a prompt action to out-of-standard events and contaminations, enhancing food safety measures.

## 5. Conclusions

We present a novel strategy that involves microbiome profiling followed by the design of PMA-qPCR-based bacterial quantification assays for bacterial indicators, enabling the rapid on-site monitoring of the production process. In our study, which used BarBQ pastrami as a model product, *Lactobacillus* and *Vibio* were identified as indicators of lactate deficiency, a finding that was further validated using PMA-qPCR. This proposed strategy is versatile and can be applied to a variety of food products, facilitating the early detection of safety and quality-related issues, and helping to prevent harmful foodborne disease outbreaks and costly product recalls.

## Figures and Tables

**Table 1:** Alpha diversity values of live microbial communities in the different meat products. Values represent average Shannon diversity indices in different batches, 5 samples tested for each. Different letters indicate significant difference (α < 0.05) via ANOVA and a following Tukey HSD test.

Products	Connecting Letters *		Alpha Diversity Mean
Sausage	A		6.17
Smoked Pastrami	A		5.81
Kabanos	A		5.65
BarBQ Pastrami		B	2.88

* Different letters indicate significant difference.

**Table 2:** Alpha diversity values of live microbial communities in the main stages along the BarBQ pastrami production chain. Values represent average Shannon diversity indices in 5 samples tested for each stage. Different letters indicate significant difference (α < 0.05) via ANOVA and a following Tukey HSD test.

Stages	Connecting Letters *				Alpha Diversity Mean
Raw material		B			4.21
Brine			C		2.81
Mixture	A				4.99
After cooking			C	D	2.76
After slicing				D	2.40

* Different letters indicate significant difference.

**Table 3:** Comparisons of Alpha and Beta diversity values for each production stage between batches with and without lactate. Beta diversities of bacterial communities are based on a weighted UniFrac measure, with *p*-values obtained using PERMANOVA tests. Alpha diversity indices were calculated using Shannon entropy, with *p*-values obtained using *t*-tests (two-tailed). Values were derived from the microbiome data presented in Figure 3.

	Beta Diversity	Alpha Diversity		
Stage	*p*-Value Obtained for Comparisons Per Each Stage	Mean Indices—Batch with Lactate	Mean Indices—Batch without Lactate	*p*-Value Obtained for Comparisons Per Each Stage
Brine	0.271	2.81	3.19	0.246
Mixture	0.004	4.99	4.06	0.008
After cooking	0.646	2.76	2.78	0.764
After slicing	0.648	2.40	2.83	0.041
One week in storage	0.702	2.82	3.16	0.271
Two weeks in storage	0.047	2.49	2.58	0.464
Six weeks in storage	0.025	2.58	2.81	0.008
Three months in storage	0.290	2.51	2.76	0.145

**Table 4:** Ct values extracted from *Vibrio*-specific qPCR. Values are averages of biological triplicates, each tested in two technical replicates, and correspond to 7.43 × 10^4^ CFU/mL, 5.05 × 10^3^ CFU/mL, 3.13 × 10^4^ CFU/mL, and <1.0 × 10^3^ CFU/mL for the raw material, the mixture with potassium lactate, the mixture without potassium lactate, and samples after slicing/storage, respectively, as inferred based on a calibration curve. Different letters indicate significant difference (α < 0.05) via ANOVA and a following Tukey HSD test.

Stages	Connecting Letters *			Ct Value
Raw material			C	19.37
Mixture with potassium lactate		B		23.01
Mixture without potassium lactate			C	20.54
After slicing/storage; with or without potassium lactate	A			25.55

* Different letters indicate significant difference.

**Table 5:** Ct values extracted from *Lactobacillus*-specific qPCR. Values are averages of biological triplicates, each tested in two technical replicates, and correspond to 7.59 × 10^3^ CFU/mL, <1.0 × 10^3^ CFU/mL (but detectable), and 5.66 × 10^3^ CFU/mL for the raw material, 6 weeks in storage without potassium lactate, and 3 months in storage without potassium lactate, respectively, as inferred based on a calibration curve. Different letters indicate significant difference (α < 0.05) via ANOVA and a following Tukey HSD test.

Stages	Connecting Letters *			Ct Value
Raw material			C	26.72
After slicing with potassium lactate	A			38.70
After slicing without potassium lactate	A	B		34.25
After six weeks in storage with potassium lactate	A	B		34.48
After six weeks in storage without potassium lactate		B		31.74
After three months in storage with potassium lactate	A			35.40
After three months in storage without potassium lactate			C	26.89

* Different letters indicate significant difference.

## Data Availability

The original contributions presented in the study are included in the article/Supplementary Material. The data discussed in this publication have been deposited in NCBI’s Gene Expression Omnibus [40] and are accessible through GEO Series accession number GSE255249 (https://www.ncbi.nlm.nih.gov/geo/query/acc.cgi?acc=GSE255249, accessed on 1 July 2024). further inquiries can be directed to the corresponding author.

## Supplementary Materials

The following supporting information can be downloaded at: https://www.mdpi.com/article/10.3390/foods13152459/s1, Figure S1: The effect of PMA treatment on pastrami microbiome profiling; Table S1: The effect of PMA treatment on DNA and live or dead bacterial amplification. (A) The effect of PMA treatment on amplification of pure DNA. (B) The effect of PMA treatment on amplification of DNA from live and dead *E. coli* cells. Table S2: All samples sequenced in the frame of the current study and their sequence data.
