# Supplementary material for: Early Detection of Food Safety and Spoilage Incidents Based on Live Microbiome Profiling and PMA-qPCR Monitoring of Indicators
Source: Foods. 2024 Aug 3;13(15):2459. doi: 10.3390/foods13152459 (PMC11311866; doi:10.3390/foods13152459)
Supplement: Supplementary file 1 [file foods-13-02459-s001.zip › Supplementary.pdf]

Setting up a DNA-based microbiome profiling protocol targeted for live Bacteria

A substantial portion of the pastrami microbiome originates in dead bacterial cells (Knipe and Rust, 2010). Therefore, we implemented a protocol distinguishing between live and dead bacteria in the frame of our DNA-based microbiome profiling process. The method, based on Propidium Monoazide (PMA) as a highly selective material that penetrates only into dead bacterial cells with damaged membrane integrity and prevents their DNA amplification, was adapted from Nocker et al.(Exterkate et al., 2015; Nocker et al., 2007; Wang et al., 2021), and applied with slight modifications as described in the materials and methods section. A complete lack of DNA amplification was demonstrated when purified DNA, which clearly reacts as-is in qPCR analysis, has been subjected to the PMA treatment prior to qPCR reaction, reaching Ct values comparable to those of the non-template controls (Table S1.A). Moreover, the protocol was approved at the cell level, presenting a significant decrease ($p<0.001$) in the DNA amplification signal of dead *Escherichia coli* (*E.coli*) cells compared to dead cell suspended in the same medium and cell concentrations yet not subjected to the PMA treatment. This amplification signal decrease is equivalent to a decline by 1-2 orders of magnitude in the cell quantity. The amplification efficiency of live cells, however, was not affected by the PMA treatment (Table S1.B).

At the next step, we turned to further test the PMA protocol on the bacterial communities present at the pastrami microenvironment. DNA samples extracted from pastrami matrices (BarBQ pastrami), with or without PMA pretreatment, were subjected to 16S rRNA sequencing. A substantial difference between the two pretreatment groups (Figure S1; $p=0.032$) was obtained along the PC1 axis, explaining 75% of the variance. qPCR targeted to the total bacteria confirmed that the PMA treatment was effective, as Ct values obtained for amplification of samples subjected to PMA were significantly higher compared to those of untreated samples (average Ct values: PMA treated samples: 25.9, untreated samples: 21.5; $p=0.0002$). This decrease corresponds to a decline of over an order of magnitude in the cell quantity, as inferred from a calibration curve carried out on *Vibrio* cells as a template. With a working protocol to analyze the live pastrami microbiome in hand, we turned to a deeper characterization of the pastrami microbiome as described in the Results section.

Table S1: The effect of PMA treatment on DNA and live or dead bacterial amplification.

(A) The effect of PMA treatment on amplification of pure DNA. 250 ng of pure DNA extracted from *E. coli* cultures or non-template controls (NTC) were subjected to PMA treatment or to no PMA treatment followed by qPCR. PMA treatment fully prevented amplification of pure DNA. The average Ct values of each three biological replications are indicated. Levels not connected by same letter are significantly different under $\alpha < 0.05$ by ANOVA and a following Tukey HSD test. Samples of DNA and NTC without PMA serve as positive and negative controls, respectively. **(B) The effect of PMA treatment on amplification of DNA from live and dead *E.coli* cells.** 10^6 CFU of *E. coli* cultures grown in LB medium and boiled at 96°C for 20 minutes or stored at 4°C for 20 minutes were subjected to PMA treatment or to no PMA treatment, followed by DNA extraction and qPCR amplification of the *uidA* gene. The decrease in the DNA amplification signal of the dead *E.coli* cells is equivalent to a decline in 1-2 orders of magnitude in the cell quantity, as inferred from a calibration curve connecting between Ct values and cell concentration detected through cell cultivation on LB Agar plates. The average Ct values of each three biological replications are indicated. Levels not connected by same letter are significantly different under $\alpha < 0.05$ by ANOVA and a following Tukey HSD test. Samples of live bacteria without PMA serve as positive control, while samples of NTC without PMA serve as negative control.

Figure S1: The effect of PMA treatment on pastrami microbiome profiling. Microbiome profiling was assessed by V3-V4 16S rRNA gene sequencing. β-Diversities of bacterial communities were clustered using principal-coordinate analysis (PCoA) based on weighted UniFrac measure. Each dot on the plot represents the total microbiota (live & dead; black dots) or the live microbiota (grey dots) of a single pastrami sample. The significant difference between the groups (p =0.032, ANOSIM R=0.81) implies that the PMA treatment is effective in profiling the live bacteria in the pastrami microenvironment.
