# Supplementary figures and images for: Early Detection of Food Safety and Spoilage Incidents Based on Live Microbiome Profiling and PMA-qPCR Monitoring of Indicators
Source: Foods. 2024 Aug 3;13(15):2459. doi: 10.3390/foods13152459 (PMC11311866; doi:10.3390/foods13152459)

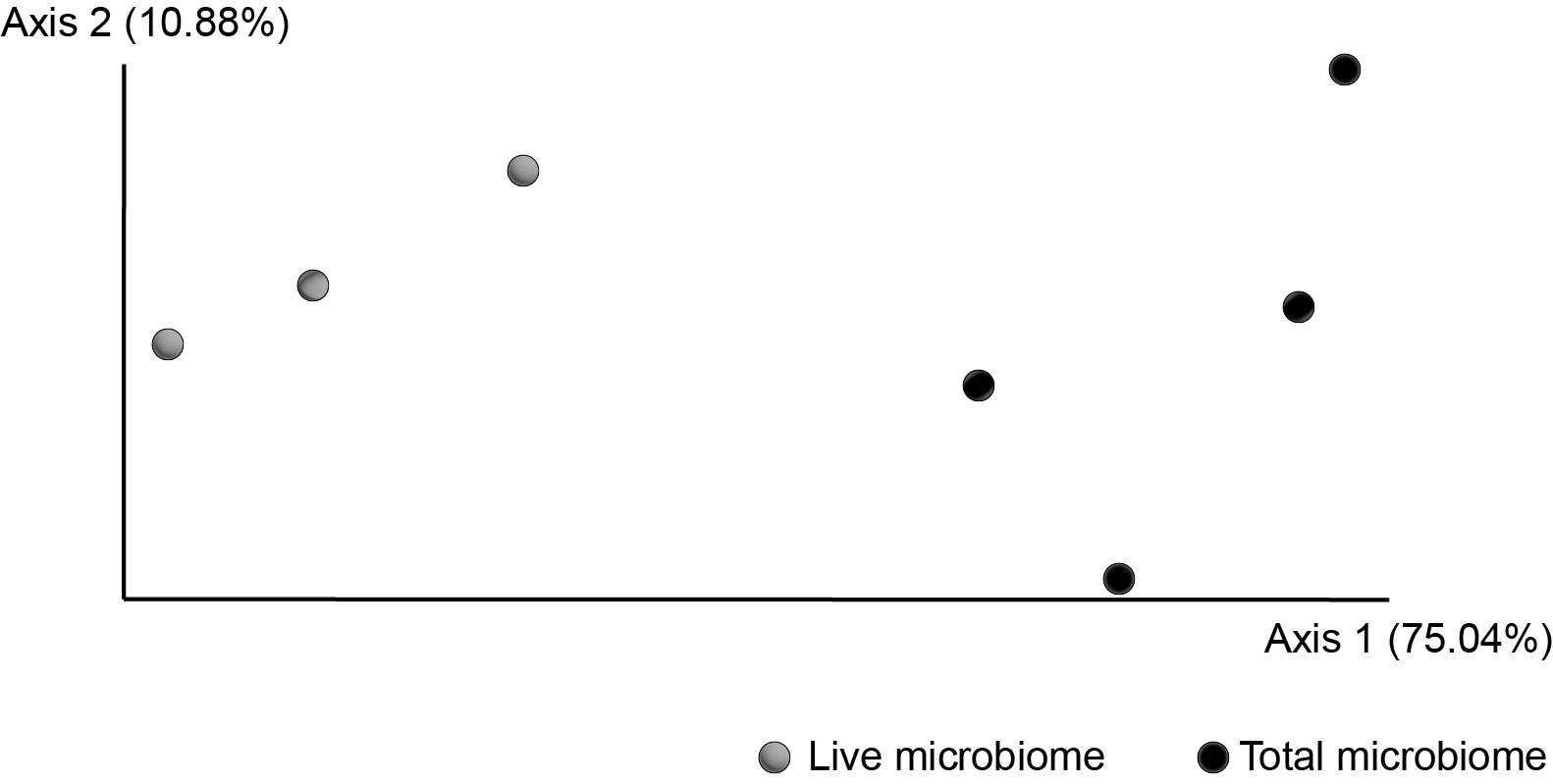

Supplement: Supplementary file 1 [file foods-13-02459-s001.zip › figure s1.png]
